# Supplementary material for: Stem-Cell-Derived β-Like Cells with a Functional PTPN2 Knockout Display Increased Immunogenicity
Source: Cells. 2022 Nov 30;11(23):3845. doi: 10.3390/cells11233845 (PMC9737324; doi:10.3390/cells11233845)
Supplement: Supplementary file 1 [file cells-11-03845-s001.zip › cells-1972139-supplementary.pdf]

Supplementary Data

Supp. Table S1: Immunofluorescence and flow cytometry antibody concentrations

**Primary Antibodies**	**Dilution**	**Company**	**Catalog Number**
Rat-human CPEP	1000	Hybridoma/DSHB	GN-ID4
Rabbit-human PTPN2	100	Millipore-Sigma	HPA046176-100UL
Mouse-human HLA-ABC	200	Biolegend	311420
Mouse-human GCG	1000	Sigma-Aldrich	G2654-100UL
Rabbit-human SST	200	Phoenix Pharmaceu-ticals	h-060-03
Guinea pig-human INS	500	Dako	A0564
Mouse-human NKX6.1	200	DSHB	F55A10
**Flow Cytometry Antibodies**	**Dilution**	**Company**	**Catalog Number**
Mouse-human FOXA2 PE	200	BD Bioscience	561589
Mouse-human SOX17 Alexa 488	200	BD Bioscience	562205
Mouse-human SOX2 Alexa 594	100	Biolegend	656106
Mouse-human TRA160 Alexa 647	100	Biolegend	330606
Mouse-human HLA-ABC	200	Biolegend	311420

Secondary Antibodies	Dilution	Company	Serial Number
DAPI	1000	Invitrogen	P36935
Anti-rat Alexa 488	1000	Thermo Fisher	A-21208
Anti-rabbit Alexa 555	1000	Thermo Fisher	A-31572
Anti-mouse Alexa 647	1000	Thermo Fisher	A-31571
Anti-mouse Alexa 555	1000	Thermo Fisher	A-21208
Anti-rabbit Alexa 647	1000	Thermo Fisher	A-31572
Anti-guinea pig Alexa 488	1000	Thermo Fisher	A-31571
Zombie Aqua Fixable Viability Kit	500	Biolegend	423102

Supp. Table S2: Western blot antibody concentrations

Antibody	Dilution	Company	Catalog Number
Rabbit-human PTPN2	500	Millipore-Sigma	HPA046176-100UL
Mouse-human HLA-ABC	100	Biolegend	311420
Anti-rabbit HRP	1000	Thermo Fisher	31460
Anti-mouse HRP	1000	Invitrogen	31430

Supp. Table S3: Differential gene expression analysis of upregulated and downregulated genes

Gene_Symbol	Average_Expression	Log2_Fold_Change	P_Value	Adj_P_Value
AC008770.4	16.9276103292279	-7.39615163730378	5.13323553328244e-09	2.32698899879208e-06
HENMT1	22.2244329093681	-7.05803948605407	1.82662732286186e-10	1.25634167523457e-07
SOX1-OT	62.8846553796748	-6.82070417351137	3.52771148549749e-05	0.0039173530578236
CHL1	37.7354908372708	-6.51485816659156	1.45642926432014e-09	7.85133462327827e-07
THEM6	88.4388675594001	-6.33578593610263	4.07979246199744e-10	2.54298563896878e-07
KCNIP3	12.1079971946242	-6.17833801281077	2.28344592140932e-07	6.996137814908E-05
IRAK4	39.7746628692618	-6.17656854141351	4.23082742525184e-14	6.49139106339025e-11
CEBPZOS	76.7311249547649	-6.11266459572748	4.44686667026804e-24	1.26710289435952e-20
QPCT	581.000221682357	-6.09394264593786	2.61422737474596e-56	2.60716896083415e-52
PHBP2	11.4243343541003	-6.0833116785505	5.43159475382799e-08	1.90067699929567e-05
ZNF841	78.7644035805555	-5.56784693682784	1.19397101799485e-30	5.9537364812313e-27
C9orf64	89.6845167261116	-5.17208005229279	1.24028453311432e-24	4.12311921624972e-21
PSCA	62.015055282846	-5.11505360784986	6.50670182525798e-09	2.76133350226799e-06
LINC01833	48.7050657590972	-5.00220954747028	2.02988369008208e-05	0.00271731946861592
ZNF781	38.3603160709087	-4.7613624798583	2.94052332474615e-16	5.86516782353868e-13
MTUS2	636.020643612133	-4.3528240845671	2.13114028207234e-39	1.4169241355405e-35
TBX1	58.3779052174914	-4.176064074434	1.89899670610291e-09	9.94572321420221e-07
AMN	219.378477713819	-4.09196422318589	5.16513737802366e-23	1.28779787677575e-19
FABP7	43.2321669325889	-4.03618423823636	5.01837872133387e-06	0.000935701496421793
TMEM45B	84.2119146766332	-3.87689327129751	1.1737221451273e-19	2.60122910074545e-16
CCDC152	28.7791743485142	-3.62262150973661	1.60041323637884e-13	2.12812282752083e-10
JAM2	78.6138613064803	-3.50639765111902	7.52140341158695e-05	0.00675774380394204
NPY	22.3037192203324	-3.4252806253089	5.01955580653424e-06	0.000935701496421793
GDF7	22.4374308454953	-3.38032292966499	1.33387467089113e-05	0.00197077512485885
GLUD2	165.294007849288	-3.15824795697689	5.39209163996166e-09	2.39001466334834e-06
ZNF501	17.0963884551785	-3.12717019066074	1.51772529765242e-05	0.00216909656488732

**ZNF844**	69.4419823533117	-3.06846553023025	2.44204733838139e-12	2.56363559007133e-09
**COL13A1**	48.6209218371613	-2.94329162577649	1.38788471967617e-05	0.00203549622196036
**STK32B**	70.6780279394179	-2.91684267863254	2.10042648731796e-05	0.00279300711440294
**CNTN2**	86.1500871466087	-2.84425131630264	3.91467720944607e-06	0.000765511290388346
**ZNF875**	426.546828321387	-2.83992930155236	6.6333763690062e-62	1.32309325056198e-57
**DAW1**	20.5320850379952	-2.83363269070895	9.46866189558136e-05	0.00807102265680623
**KLK13**	130.288525812354	-2.80807845016074	0.000133712813518276	0.0102578299170598
**ADGRB1**	82.7241568817753	-2.76787088675132	6.58357800204366e-05	0.00617376687865502
**PUS7L**	153.19874669085	-2.7533375010483	1.38511045267416e-12	1.53485628272438e-09
**LINC01535**	13.3847195540517	-2.73658900678414	2.26932890443288e-05	0.002864812299229
**TRIM58**	30.7682757754058	-2.71331709117127	0.00016417981376709	0.0117373855390623
**TF**	461.221070033873	-2.64430477693544	1.49399119506309e-10	1.10367216210105e-07
**ZNF283**	49.6713828531055	-2.6281048255158	3.80881368453397e-12	3.6176475119864e-09
**SOX2**	571.923332121034	-2.61756685805309	3.45579690575688e-06	0.000718013802939861
**KLK10**	126.584306411394	-2.61629991468919	7.19288146047081e-07	0.000196533169329522
**TRABD2B**	224.980499285663	-2.57900512903945	5.24942319060342e-05	0.00536948692101414
**H2BC14**	45.1993153315963	-2.52206269418926	6.82867700437892e-07	0.000191837734548369
**SLIT1**	1950.54526575647	-2.49766875388471	0.000154868622749717	0.0113985592227522
**CYP26B1**	89.2254716779295	-2.49341587022212	0.000214966201529005	0.0142449031750749
**MIR124-1HG**	94.7558873869623	-2.47192565067249	2.23050186954164e-06	0.00049180221606157
**AL137786.1**	12.2593830962957	-2.41176617632217	2.34497676078855e-05	0.00294169223086091
**CLCN3P1**	30.1493336390662	-2.38617426021343	1.71802361440643e-06	0.000398461616429658
**BMPR1B**	63.4261528621762	-2.34733396335961	2.3929385215376e-05	0.0029645684317136
**GRIA1**	153.440788693016	-2.30587223873594	1.10885788971595e-05	0.00174151806836805
**LINC00665**	190.691029814776	-2.30054755859959	1.30688940701946e-14	2.17226800936751e-11
**VWA3A**	96.4253677697378	-2.21855345367053	1.6011131516688e-10	1.14056439011378e-07
**GALR1**	63.9096649099122	-2.17565896591353	0.000196458560641732	0.013419734419726
**TCEAL5**	287.188954752021	-2.14116443032159	3.47636926252874e-11	2.88915255459993e-08
**LINC01882**	15.6418226754415	-2.13668998729675	0.000191820303346306	0.0131479304829739
**FAM66B**	70.5809994082742	-2.12621438515264	2.29194676942571e-09	1.11500415275525e-06
**FABP3**	247.000143616805	-2.0874051423977	5.54793259210174e-05	0.00555611031389358
**ST8SIA1**	87.207918818387	-2.07943556716369	4.28774137224221e-05	0.00462288050868881
**AC007608.3**	20.9052968988956	-2.05894256120298	1.61041371595812e-05	0.00226206422383807
**PNCK**	16.935839745172	-2.05654237894118	0.000147786565174561	0.0111657228370144
**VASH2**	51.9077413430709	-2.04896152254863	0.00013368089364277	0.0102578299170598
**PRDM8**	28.4296028476535	-2.03280615040869	0.000166953814694995	0.0118649665394584
**RGS5**	287.822997382032	-2.02419574702978	2.36995881069213e-09	1.12550472471584e-06
**CLDN19**	67.1188435830853	-1.9461166670589	0.000119486519792281	0.00959150550399578
**ELAVL3**	187.532017158908	-1.92830416591448	0.000110543824908123	0.00903650463777628
**RGS22**	29.374154953076	-1.91061145595221	3.19531376559337e-05	0.00362123456639349
**ARHGAP19-SLIT1**	72.9616357587185	-1.90041831758642	0.000126944822494053	0.00985230128197038
**GRIK4**	126.659870439044	-1.85905176823613	3.01092532146466e-05	0.00347144025791527
**PTGIS**	94.9829607943162	-1.85698029762023	1.99193702249305e-09	9.94572321420221e-07
**SYNDIG1**	63.4566782425514	-1.84169563820344	2.46995843772767e-07	7.35310313416658e-05
**SDK2**	1421.98065996761	-1.78959782951525	3.12584288901761e-11	2.7107853158411e-08
**EPHB1**	269.765921624948	-1.78128638421785	4.95039452891635e-06	0.000935701496421793
**CDHR3**	641.412784962643	-1.76525518080253	1.30170162253816e-05	0.00195451768399138
**LRP8**	472.415408187504	-1.69875760274219	7.59297616010876e-10	4.20693062470915e-07
**CAVIN4**	49.5026436411105	-1.69162116740513	5.77192814490774e-06	0.0010562098970489
**SNORD17**	2593.46180733423	-1.67745538114853	8.52134155893746e-06	0.00141638898945472

**ADAM23**	142.682662105269	-1.66664585392292	3.52470584435706e-06	0.000722490392190181
**APBA2**	365.663991948344	-1.61253562861292	2.20215485758097e-06	0.00049180221606157
**CFAP74**	94.3688459628154	-1.60306640629755	0.000173153053900516	0.0121581360523346
**ENO4**	55.0787331657322	-1.59242608174577	0.000221939684838737	0.0145119552285874
**EFR3B**	279.549450164911	-1.5838699407528	8.40595255224786e-05	0.00735373375469894
**EFCAB12**	108.297069902356	-1.56617776305814	3.71945082293423e-05	0.0040987937079694
**TSPAN18**	467.69395989169	-1.55143466640765	1.34617932693837e-12	1.53485628272438e-09
**NRIP3**	68.8488449812889	-1.54451659099122	2.48235587302584e-05	0.00301908964898619
**PLTP**	603.941737527216	-1.53003826765601	1.86814384207155e-08	7.16576866806907e-06
**INSYN2A**	73.0928609446169	-1.5238510761745	0.000108237540474816	0.00893601055463958
**SLC35F1**	76.1206838427196	-1.50592556827591	0.000167154095938424	0.0118649665394584
**PNPLA3**	188.639996927308	-1.49958783483527	2.40098668016755e-13	2.99313002016387e-10
**CDH26**	115.350570745589	-1.49392742129265	4.07948747792311e-06	0.000789994730433537
**TAFA5**	229.094718550793	-1.46842836817791	6.29942526728723e-07	0.000182099038233784
**PCSK9**	252.629818439525	-1.46353153152925	0.00015351688955631	0.0113985592227522
**PCDHGB7**	163.472778642781	-1.46184914248256	2.71162252253576e-05	0.00318153075497049
**ATP8A2**	127.769132657606	-1.43948790304217	0.000122828172801065	0.00976068021788865
**GRIK3**	1727.50652298755	-1.39657416566537	0.000200755128026782	0.0136199380395313
**CDH7**	65.5925124931835	-1.38682777843488	3.91779387936083e-05	0.00429364377569952
**GPR162**	92.0389754486906	-1.35544940171381	5.97045974443618e-06	0.00106409105299985
**SLC13A3**	47.8053778697953	-1.35401978045706	2.97949727693013e-05	0.00347144025791527
**CFAP92**	226.452725601287	-1.32565999544946	1.30323168561797e-06	0.000322408125777479
**AFF3**	325.909893342799	-1.31435817482215	8.06261313292951e-05	0.00717932506917018
**HOMER3**	126.022044252044	-1.30317769206358	0.00015269463541478	0.0113985592227522
**PDE11A**	76.7637593054119	-1.26126624883777	1.72973878647568e-05	0.00239592846076694
**TMEM178A**	74.5804452578439	-1.25503629210251	8.20921245870666e-05	0.00727737563117169
**CIT**	769.292513583339	-1.24672905774164	0.000105742002787095	0.00879360998598132
**SPAG8**	48.7123053249938	-1.24139344449071	5.94830742803282e-05	0.0057316396115721
**AK5**	361.137328323812	-1.22066379283883	7.18395714179536e-05	0.00661977555674585
**CRB2**	977.761462919898	-1.20973196224931	0.000161033546371651	0.0116375909997426
**LINC01197**	81.5347911459607	-1.19684593922274	5.58777754504273e-05	0.00555611031389358
**LRRIQ1**	125.655441453368	-1.17273492982266	2.24375823030196e-06	0.00049180221606157
**GALNT16**	300.865780654425	-1.16135926421916	6.66393619432426e-07	0.000189884101902845
**TMEFF1**	294.89596578478	-1.15476803226501	7.43885509862046e-10	4.20693062470915e-07
**POU2F2**	66.580024788132	-1.13094933418088	0.000169958455616878	0.0119787680414638
**GLB1L2**	240.106799463853	-1.09132361427727	7.45552993379869e-05	0.00672886878097505
**ZFP82**	120.770546234535	-1.06629333268348	6.80541531956475e-06	0.00117017943072447
**STARD9**	610.759849242279	-1.04514628237199	4.09671421114397e-08	1.48569203009959e-05
**FAT3**	3819.72464744818	-1.03765616904463	1.15639644519088e-15	2.09686213597976e-12
**KLHL14**	57.8631778470166	-1.03015940168751	2.44573066384386e-05	0.00299279409944968
**TIMELESS**	1058.80714228094	-1.02961955429386	7.20613246121756e-08	2.39555863452409e-05
**FAM27C**	144.497796722378	-1.01830841573925	0.000160195446475485	0.011619121365091
**PER3**	312.138818111883	-1.00026857968478	1.32967411905676e-06	0.000323435121691539
**ARHGEF4**	393.903616096281	-0.98657544115277	9.42751048246956e-06	0.00154132068920769
**CAD**	1925.62838252244	-0.42510279831855	9.61772563802583e-06	0.00154705770625857
**CD63**	4231.97757539401	0.43026675329535	6.16784247884919e-06	0.00108870607153209
**DOK4**	1382.93051899788	0.58644953358132	0.00015425526529879	0.0113985592227522
**MAP3K13**	467.404688463618	0.62595807656109	5.90013125161823e-05	0.00571281640508627
**TMEM59**	4231.27125936604	0.64128846244713	1.57114863972981e-05	0.00222256246581921
**MYO3A**	2514.93741799287	0.71208607816489	6.30105424445592e-06	0.00109917649954437

**CLU**	22723.4887331088	0.91428696107348	4.31956280604344e-05	0.00463215052308293
**CCDC68**	210.911022894206	0.99157808388485	7.2019016134255e-05	0.00661977555674585
**KRT8P46**	135.000746511139	1.02163650895731	0.000207393559537607	0.0138432812990456
**CTSZ**	9169.90692838244	1.07617418292432	7.62419529455054e-07	0.00020276293246014
**SMIM6**	370.074559747987	1.10029612900564	1.73397533140045e-07	5.48982094604975e-05
**DMTN**	1291.52579946529	1.10256211272733	7.39294232624936e-05	0.00670271034724408
**TMEM238**	414.868084386097	1.1210895561402	0.000108418457546515	0.00893601055463958
**AC012101.2**	311.850458045992	1.14643663230266	2.42774865750229e-05	0.00298912806929264
**ALDH1A1**	11238.7957971455	1.17028605182656	1.42800092648011e-05	0.00207904426858192
**CDH10**	591.751308877031	1.17979430246455	3.12351066480867e-07	9.16199172356966e-05
**LINC01036**	67.549546747744	1.23340190567192	0.000112587546630062	0.00916600491870697
**SKOR2**	185.695663007126	1.24817383027903	2.83493491095929e-09	1.31501422637195e-06
**EGFL7**	1614.28459159702	1.25242377454946	1.22409524469887e-24	4.12311921624972e-21
**FA2H**	207.004769215174	1.25725891067001	6.33737578700502e-06	0.00109917649954437
**GDA**	422.787589799994	1.25957341641157	0.000105809004142962	0.00879360998598132
**BIK**	189.755426037418	1.26933683752494	1.65842294878039e-06	0.000389163578074985
**VWA5A**	2027.08277524063	1.27767806396723	6.28669096531716e-11	5.01577351976865e-08
**EGFR**	2616.65661040266	1.2863138664191	1.99452987349889e-09	9.94572321420221e-07
**TTC22**	341.340189287422	1.31526950828773	0.000119791761620071	0.00959150550399578
**ZNF69**	103.8825355431	1.33155264424366	6.66417963058594e-08	2.25294452392656e-05
**C2CD4B**	970.237680735714	1.33543439622759	1.41113193779913e-13	2.0104598308101e-10
**TMEM236**	1700.24767375668	1.33596018436942	0.000109033482945467	0.00894971955074193
**TMEM61**	201.47753364237	1.34361843409112	3.53516269130777e-05	0.0039173530578236
**BDKRB2**	668.954618464325	1.37551213782597	1.10278489336655e-05	0.00174151806836805
**NCKAP5**	2158.2329993646	1.42923974776548	2.99416901164916e-05	0.00347144025791527
**HS3ST3B1**	398.090706263412	1.44849034193904	0.000126798792863621	0.00985230128197038
**C2CD4A**	1453.06590003316	1.50895898957441	3.95810562067319e-10	2.54298563896878e-07
**PCDH10**	917.871615080274	1.53263080206173	5.09107465749352e-05	0.00526148057608113
**CNTN1**	1848.24633318589	1.54626584773342	1.79091042736612e-07	5.58148427878823e-05
**IL1R1**	1174.74606267636	1.54938894922828	5.63877585752549e-06	0.00104139836346485
**GRPR**	357.942743727892	1.59845827988076	2.38442630593704e-05	0.0029645684317136
**LINC00643**	830.468368781083	1.60212909847579	3.96608047276246e-05	0.00432281098960218
**ANKRD53**	152.298616418279	1.61234929493344	5.60105553852915e-05	0.00555611031389358
**PON3**	34.5747344986133	1.66666800772157	9.39936329851868e-06	0.00154132068920769
**BTBD17**	907.005554160403	1.72398600739252	2.67126183887601e-08	9.86684974781869e-06
**ZP1**	30.5557124338402	1.75780280091701	3.09687607743044e-05	0.00352973087088157
**CALB1**	97.3047216267614	1.77243461465301	2.01168338108377e-06	0.000455966326353373
**SLC38A4**	1452.97722572857	1.79394801532308	4.41777508921889e-10	2.67021036150182e-07
**AC093898.1**	107.108144153928	1.80464496843297	3.99689567630366e-12	3.62373096179786e-09
**SYT16**	351.410901121448	1.81185807427479	0.000136850418643547	0.0104583082385601
**SCIN**	199.634430306675	1.81253038677059	3.93478927626333e-10	2.54298563896878e-07
**CYP1A1**	4328.65786077582	1.86859998690415	1.32188916938754e-08	5.49300028595914e-06
**AC012645.1**	26.4691397446541	1.93016904429797	1.19333393890861e-05	0.00184339835200186
**GAS2L3**	1263.98485482806	1.95015348034589	1.43978623862278e-07	4.63193166380161e-05
**MLN**	111.727169898469	1.97705570330448	9.3565181559145e-05	0.00800966142222621
**FGF18**	90.6144331921781	2.02565335536336	4.39868944318282e-08	1.56671892203079e-05
**PTGER3**	1479.97381343113	2.16476573073557	0.000220232079436714	0.0144498324225155
**CALHM2**	27.9883106659349	2.29953126267851	1.52247828679547e-05	0.00216909656488732
**AL355102.4**	62.2049638694738	2.63458146057286	9.5494402698009e-06	0.00154705770625857
**LINC01451**	20.6396015723203	2.73001046394698	1.32003453341695e-05	0.00196488125399511
**SPX**	96.9916767659345	3.59380045569277	7.24322603545142e-05	0.00662721956436303

**CEP250**	2089.34623470659	-0.497747643905468	5.80056337099108e-05	0.00566611436424441
**CECR2**	933.617377286061	-0.515135543768792	0.000208650982267278	0.0138725083076771
**WDR19**	665.11030940063	-0.517897518834272	3.57369354556838e-06	0.000722490392190181
**LIMD2**	430.085214007991	-0.537450503220005	2.80614766674487e-06	0.000608385014792316
**NFATC4**	396.602308645138	-0.540938559210074	0.000187926325363419	0.0129254430541336
**FYN**	1319.2931636759	-0.549054960190743	0.000118028094320999	0.00953112700132242
**EPB41**	1740.43228119262	-0.562649734523383	1.86333247872431e-05	0.00254561846716679
**CEP41**	315.128166645493	-0.574323503383571	0.000103986034105238	0.00875149973106783
**ZNF75A**	232.736043849054	-0.598533807784775	1.90281523832858e-05	0.00258187433630625
**MICAL1**	1842.26844033216	-0.605415635964751	1.93743673140979e-05	0.00261108871923647
**CAMSAP2**	823.824054665051	-0.620308385762817	1.81295631845399e-06	0.000415646284228544
**PLCG1**	2149.50854664974	-0.623864112093955	3.24034164113417e-12	3.23159271870311e-09
**FADS1**	3636.18795746839	-0.638626792089584	2.92561820621313e-06	0.000620791284480075
**MSI1**	1197.22850852444	-0.666172178400032	6.96981176325777e-06	0.00118820397803367
**THBS3**	388.619502710537	-0.674590633050501	2.16652757104644e-05	0.00284299729816396
**PHF21B**	418.214328227067	-0.723527424525908	0.000176511490146803	0.0123101335051333
**ZMYM3**	982.304320153659	-0.727283442354117	5.57944450273049e-05	0.00555611031389358
**ENAH**	1686.65818901823	-0.742829411687303	0.000144074652376884	0.0109266654612522
**C20orf96**	362.415969944647	-0.755288580528666	1.37320960694328e-06	0.000330000467711936
**ZNF615**	133.326188524529	-0.763419994342447	4.68523352494563e-05	0.00489739592725707
**PPP2R3B**	710.723392037883	-0.765624147096903	0.000163964337555469	0.0117373855390623
**CROCC**	1175.23179251627	-0.774529014740255	1.2106947965118e-05	0.00184339835200186
**DDAH2**	456.288639990493	-0.802038471063271	5.16626907826511e-05	0.00531167025954
**RPGRIP1L**	291.143399572386	-0.831876556260702	1.30327309721675e-05	0.00195451768399138
**PTPN2**	486.539380816153	-0.838380429588731	7.92543888510279e-11	6.08003092316386e-08
**ZNF107**	179.018733412328	-0.855155615979657	2.22849058711307e-05	0.002864812299229
**CFAP43**	137.077836028583	-0.868371783018719	6.5513827488739e-05	0.00617376687865502
**MPP3**	110.653621259928	-0.871717188908051	6.60443327781958e-05	0.00617376687865502
**AL513548.4**	59.9700053714785	-0.929158754503423	0.000150926472085583	0.0113599223102606
**KCTD15**	308.510444081757	-0.936189298634179	0.000229771134413292	0.0148799189837906
**AC107871.1**	93.7469028609015	-0.944790575143065	8.4728007103711e-05	0.00735555115427981
**GPR173**	206.488605668234	-0.960320409447778	0.000115558159895364	0.00936960592387373
**ERC2**	166.937011408034	-0.979008583271439	9.99031836464025e-07	0.000252236569748246
**ASAP3**	503.617796472737	-0.987623803023967	0.000168973913120302	0.0119516087627573
**ZNF527**	96.1081924184032	-0.997881993381917	4.4007903065632e-05	0.00465649558306719
**EIF5**	5121.14945437085	0.340821580233486	0.000216714795876081	0.0142834097047447
**UBE2W**	577.830833252533	0.407082902884412	0.000197318809853635	0.0134324948168622
**LGMN**	1360.80470913065	0.415183808753404	5.47225104001789e-05	0.00554058473320796
**RAB11A**	3317.15889194922	0.416782514244983	1.20967958555327e-05	0.00184339835200186
**NFE2L2**	1314.86625484967	0.429851706486932	7.38391587523885e-05	0.00670271034724408
**SCP2**	1413.78960158704	0.436274816333457	0.000125791824264924	0.00983954990539441
**ITM2B**	4912.2521791814	0.443336500722429	0.000125793904836838	0.00983954990539441
**DAP**	2901.69312947811	0.448055452444758	2.56253230093614e-05	0.00305926700012906
**BCAP31**	1726.59208759253	0.451589948061293	5.62686394969669e-05	0.00555611031389358
**SRPRB**	1210.06696357008	0.452604654718301	0.000205510956237189	0.0138017560037272
**TMEM167A**	2697.26497381832	0.453701263602191	0.000207517352271866	0.0138432812990456
**CAPN2**	6486.86670444671	0.460827191376836	2.23577101820787e-05	0.002864812299229
**RNASEK**	2965.40345944679	0.474265233426514	1.39874224019423e-06	0.000332134675272788
**GSDMD**	760.963599925207	0.478131513310616	0.000178346379890658	0.0123947626944218
**SEC24D**	3450.98473074605	0.493564350006012	1.32000449651681e-07	4.31619830943023e-05
**YPEL5**	1422.58364175868	0.496160432398143	0.000128282817784278	0.00991755458730698

**VASP**	1190.82694127572	0.497399420914438	0.000123551205191485	0.00977917594741808
**TMTC1**	2799.23667280133	0.497713012655192	2.57674148211011e-05	0.00305926700012906
**KRT10**	374.100831273322	0.499314612281675	5.82349064810039e-05	0.00566611436424441
**NCOA6**	3137.45178583311	0.500809020288594	0.000203309451153162	0.0137464756362744
**RAB11FIP5**	766.328890994169	0.509833166802542	3.03591650696525e-05	0.00348013739355913
**TUSC3**	3342.44596110161	0.516533367724699	4.75256313069461e-06	0.000911486771200334
**TMED3**	3016.59668421727	0.516753313273698	0.000224713143827288	0.0145997666670328
**PERP**	2232.62514795736	0.521923951903104	1.30928798696359e-06	0.000322408125777479
**DNAJC3**	2144.84967934221	0.526355295565942	0.000180744240935133	0.0125177938530978
**SOWAHB**	318.476260566584	0.527031680739193	0.000120218408502905	0.00959150550399578
**SAMD4A**	1421.0761724219	0.528600884467419	0.00018544959069198	0.0127992302281738
**GPX7**	663.367767608937	0.534722741514324	3.50492834001341e-05	0.0039173530578236
**SETD7**	1253.70183086625	0.535616056374854	6.62381486028363e-05	0.00617376687865502
**UQCR11**	880.83452400842	0.540756178835581	8.95137323574321e-05	0.00772918140952961
**ASAP1**	4038.782662012	0.543608969064408	0.000156968599460747	0.0114726140503245
**PDE3B**	3530.13331411808	0.543698626577196	0.000104886514321084	0.00879019501953088
**CASP3**	772.867473772907	0.548360592002552	5.97504251158043e-06	0.00106409105299985
**C11orf24**	986.985080963047	0.549906438448025	0.000152707050330297	0.0113985592227522
**ITCH**	1857.84666257036	0.551236624747489	1.79392564515844e-08	7.01600802320203e-06
**SSR4**	3504.13897109569	0.559321514039543	2.24720456241084e-05	0.002864812299229
**GPD2**	1068.02519150389	0.577392431875913	3.86865208790247e-06	0.000764001332131709
**GAB2**	1380.10722681697	0.588462719499591	1.50498658509296e-05	0.00216909656488732
**CYBA**	1137.40192196993	0.594030346303346	0.00017372248946733	0.0121581360523346
**TP53INP2**	2602.67007245458	0.598274433968179	7.13166988778574e-05	0.00661619942240811
**ATP6V0B**	1301.45793354913	0.598481425131574	0.000222634026869936	0.0145119552285874
**PRKD3**	1059.3561406435	0.604973035078408	2.11970263039959e-05	0.00279997275933445
**DHRS7**	1798.82794412276	0.608613560260215	0.000139922581408027	0.0106522740792538
**P4HA2**	1785.85160577395	0.610706129055727	3.40067815042142e-06	0.00071399922514006
**CIB1**	356.388507468355	0.625029237105077	8.4580288252312e-06	0.00141638898945472
**FKBP2**	1104.42852225164	0.635699041979698	5.92870484638003e-08	2.03886115286028e-05
**ELL2**	5693.46113107409	0.657798582412891	2.03184453677009e-08	7.6466360623427e-06
**MTFR1**	586.569707157251	0.660996623298216	3.58801801940312e-06	0.000722490392190181
**MON1B**	1904.85908296856	0.663856753281837	9.93595174291949e-05	0.00839756328238441
**MGST2**	372.013146855127	0.671791835544223	4.07489103276523e-05	0.00441727046410517
**SMARCA2**	13572.7885605972	0.674616788252847	7.30169311974379e-07	0.000196810231035689
**TLCD2**	348.654707052347	0.687108946305447	8.48178464596589e-05	0.00735555115427981
**CBR1**	713.464809504068	0.687380200802609	5.03736917002928e-05	0.00523309195132312
**TTC39A**	1869.74605804322	0.688825008826288	0.000163942214376666	0.0117373855390623
**IL10RB**	223.659453278399	0.691255266827149	8.36959352991524e-05	0.00735373375469894
**SASH1**	1537.10553905624	0.691530121699669	5.30696031111822e-05	0.00540064440640632
**GRPEL2**	338.198965243065	0.693750994079816	3.4976504533197e-05	0.0039173530578236
**TIPARP**	1320.13442714781	0.715550346051426	3.62223198731666e-06	0.000722490392190181
**MYLIP**	396.085756172869	0.724911768133687	0.000154738287525345	0.0113985592227522
**MPP2**	872.223785803888	0.732158444627524	1.69707892721329e-05	0.0023671284113424
**AGPAT2**	879.116067752332	0.735624960617983	2.66569197907271e-05	0.00314614746831859
**CST3**	5338.87217296872	0.735929330364531	0.000205273279385237	0.0138017560037272
**C4orf48**	362.878075534512	0.737910496529894	9.27182476880858e-05	0.00797137141545931
**CDS1**	532.627563431706	0.745749825590034	2.19554866797444e-05	0.00286224926349138
**HM13**	3595.20834073965	0.762890840514099	7.18694852032962e-07	0.000196533169329522
**VAMP8**	471.809438973302	0.767480416255672	0.000158158691293131	0.0115132600603386
**MAPK13**	2725.36846933216	0.777249727433374	8.86180157740653e-07	0.000229555187354481

**IL17RA**	739.562292653066	0.783692011312545	1.05997642540608e-05	0.00169138318249198
**PAK6**	273.584253525148	0.787461657207155	4.41230154015692e-05	0.00465649558306719
**DOK6**	1808.96171362588	0.799951619618196	4.35154948440555e-05	0.00464149764791193
**ANKS4B**	2262.13856378564	0.806303665257318	7.88209333057703e-07	0.000206863465225907
**FOXO1**	1711.75507390885	0.807181713823938	8.33753739989529e-05	0.00735373375469894
**GAREM1**	2067.1374451853	0.816638474455278	6.17611799158602e-05	0.00592254083943148
**CACNA2D1**	5675.49671534187	0.842958198205838	1.86102542304589e-05	0.00254561846716679
**FFAR1**	792.698209576178	0.845956605360298	5.66338444183846e-05	0.00556462394467537
**AL035071.1**	206.890398225742	0.848268188484389	6.30859437702672e-05	0.00602063270067823
**ATP1B1**	3342.87248487682	0.860040649072349	5.2520709993019e-10	3.08111200447282e-07
**PLIN2**	2804.53287652331	0.869257605501486	9.52964958137663e-05	0.00808844215106972
**SMG8**	360.490084359587	0.870295027544105	2.31497591388714e-07	6.996137814908E-05
**F3**	917.506165898506	0.872131210201091	8.03444739422415e-06	0.00135809396377284
**CNNM1**	442.135286666705	0.889062281626063	6.34255438887451e-05	0.00602421856383291
**SLC43A2**	3691.04060724946	0.913246196127101	4.68967523366139e-05	0.00489739592725707
**SLC7A14**	1939.15521623731	0.925889973342565	5.96991506097722e-06	0.00106409105299985
**TRIB1**	818.235787562682	0.928422185095651	2.55664905275011e-05	0.00305926700012906
**ZFP92**	90.6592276274678	0.934596065459751	7.57326323029628e-05	0.00677382548840761
**SH3BP5**	1396.35644241654	0.939991803887889	2.25531255070538e-05	0.002864812299229
**PDE5A**	865.289277700832	0.944693862358633	0.000157025149691095	0.0114726140503245
**RAB31**	322.878598092589	0.945733378118101	6.38726090131617e-09	2.76133350226799e-06
**TMPRSS2**	780.880799934071	0.945802019062892	1.49328260195091e-08	6.07857444459445e-06
**MET**	2495.03103249175	0.962626950784099	9.8602052791404e-07	0.000252143146791967
**PYCARD**	94.657680741809	0.968183008066486	0.000124081149640508	0.0097823028092078
**GPR142**	674.395741266071	0.970875930299713	2.50350828525295e-05	0.00302636219743365
**GNAS**	39101.3162437221	0.976643388762739	2.8853725631024e-06	0.000618834851006887
**SHROOM1**	168.715645907283	0.977429654501559	1.18351227195308e-05	0.00184339835200186
**RGS6**	684.402523630763	0.982914320926207	0.000216979501681422	0.0142834097047447
**ABCG1**	290.441512263076	0.986725884900798	1.73823741025047e-08	6.93417667697118e-06

Supplementary Figure S1

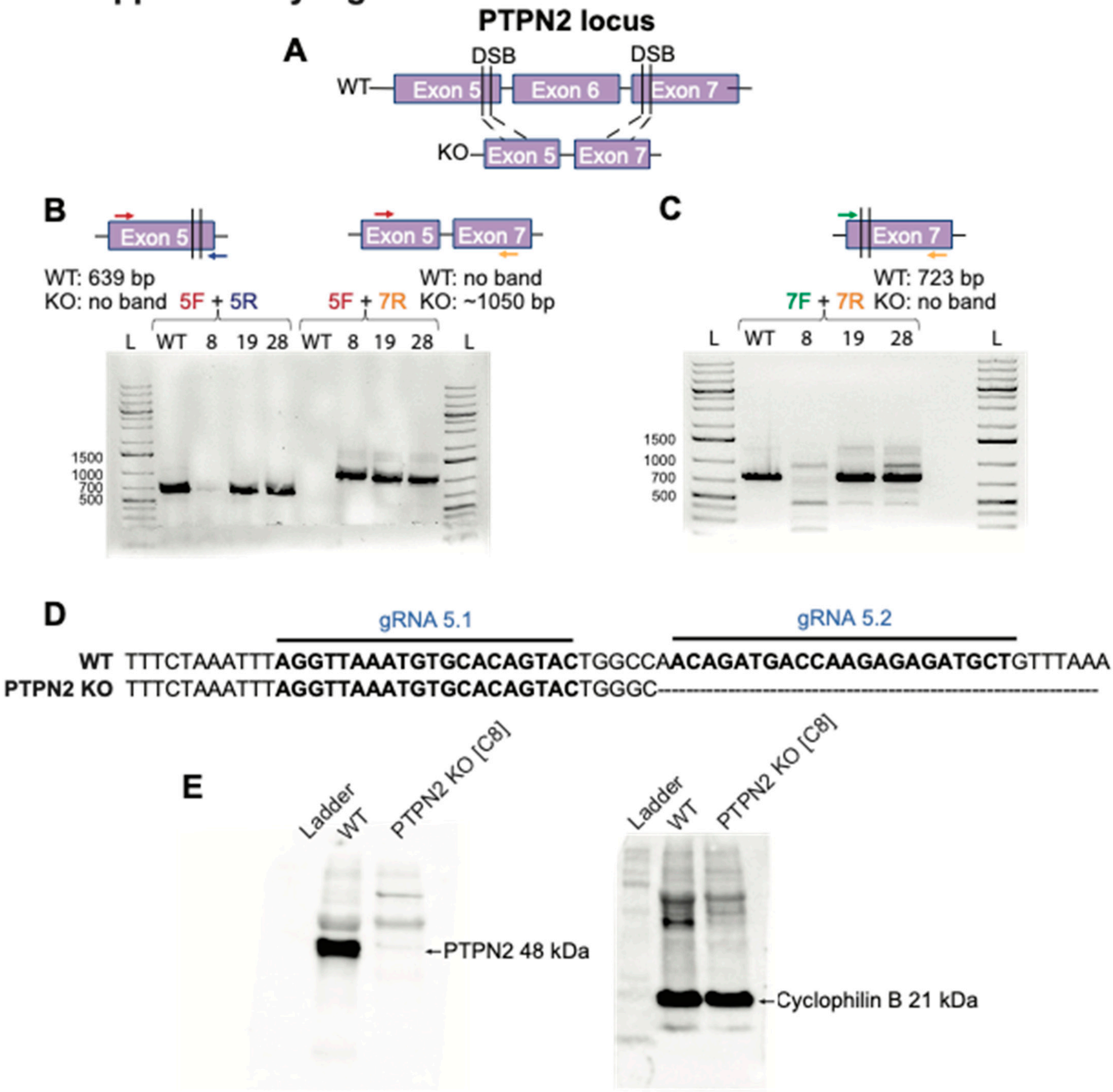

Supplemental Figure S1: Generation of a CRISPR mediated knock out of *PTPN2* in human pluripotent stem cells

A: Schematic of CRISPR-mediated disruption of *PTPN2* in hPSCs at the functional domain between exons 5 and 7. Two gRNAs targeted the functional domain of *PTPN2* at exon 5 and two at 7 inducing a large frame shift mutation in exon 5. B: gDNA PCR amplification of clonal WT and PTPN2 KO clones of interest (C8, C19, C28) hPSC lines employing primers that will amplify intact, unedited DNA of exon 5 with the forward primer in exon 5 and the reverse primer in exon 5 and employing primers that will only amplify after successful deletion of DNA piece spanning exon 5-7 with the forward primer in exon 5 and the reverse primer in exon 7. A 639 bp band amplified in WT and no band amplified in the PTPN2 KO clones for the 5F and 5R primers. A 1050 bp band amplified in the PTPN2 KO clones and no band amplified in the WT for the 5F and 7R primers. C: gDNA PCR amplification of clonal WT and PTPN2 KO clones of interest (C8, C19, C28) hPSC lines employing primers that will amplify intact, unedited DNA of exon 7 with the forward primer in exon 7 and the reverse primer in exon 7. A 723 bp band amplified in WT and no band amplified in the PTPN2 KO clones for the 7F and 7R primers. D: Sanger sequencing of exon 5 in WT and PTPN2 KO clone 8 after CRISPR-Cas9 double stranded break showing large deletion of bp in clone 8, clones 19 and 28 were excluded due to in-frame deletions. E: Uncropped representative western blot of PTPN2 protein expression in WT and PTPN2

KO hPSC. Bars indicate protein expression in WT hPSCs at 48 kDa and no expression in PTPN2 KO (C8) hPSCs. (n=3 independent analysis) Normalized to endogenous control protein cyclophilin b at 21 kDa.

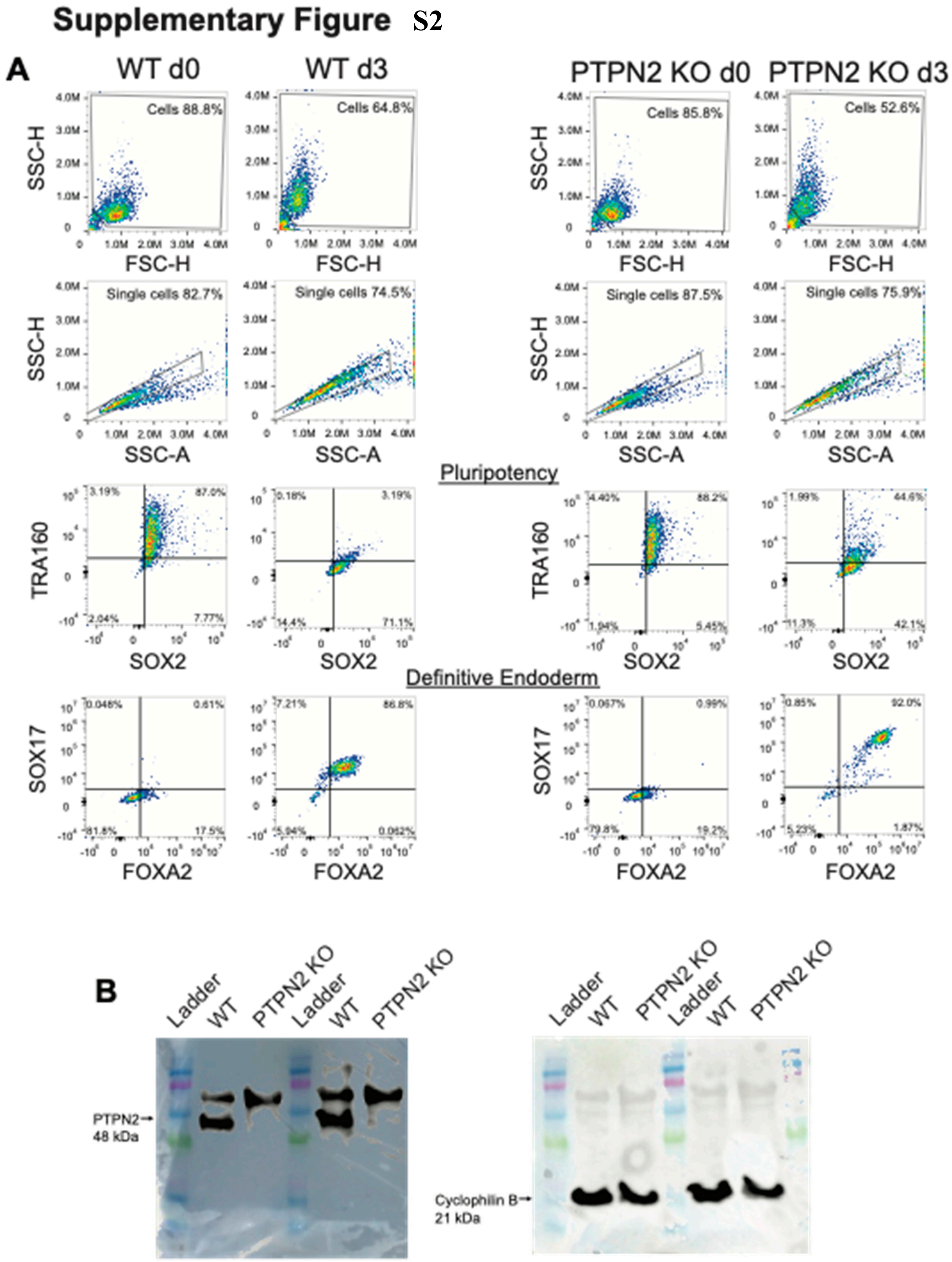

Supplemental Figure S2: WT and PTPN2 KO sBCs demonstrate similar phenotypic features during direct differentiation

A: Representative flow cytometric gating strategy throughout differentiation of pluripotency markers (SOX2 and TRA160) and definitive endoderm markers (SOX17 and FOXA2) at day 0 (d0) and day 3 (d3) for WT and PTPN2 KO sBCs. B: Representative western blot of PTPN2 protein at 48 kDa from GFP+ sBCs from WT and PTPN2 KO hPSC. (n=3) Normalized to endogenous control protein cyclophilin B at 21 kDa.

Supplementary Figure S3

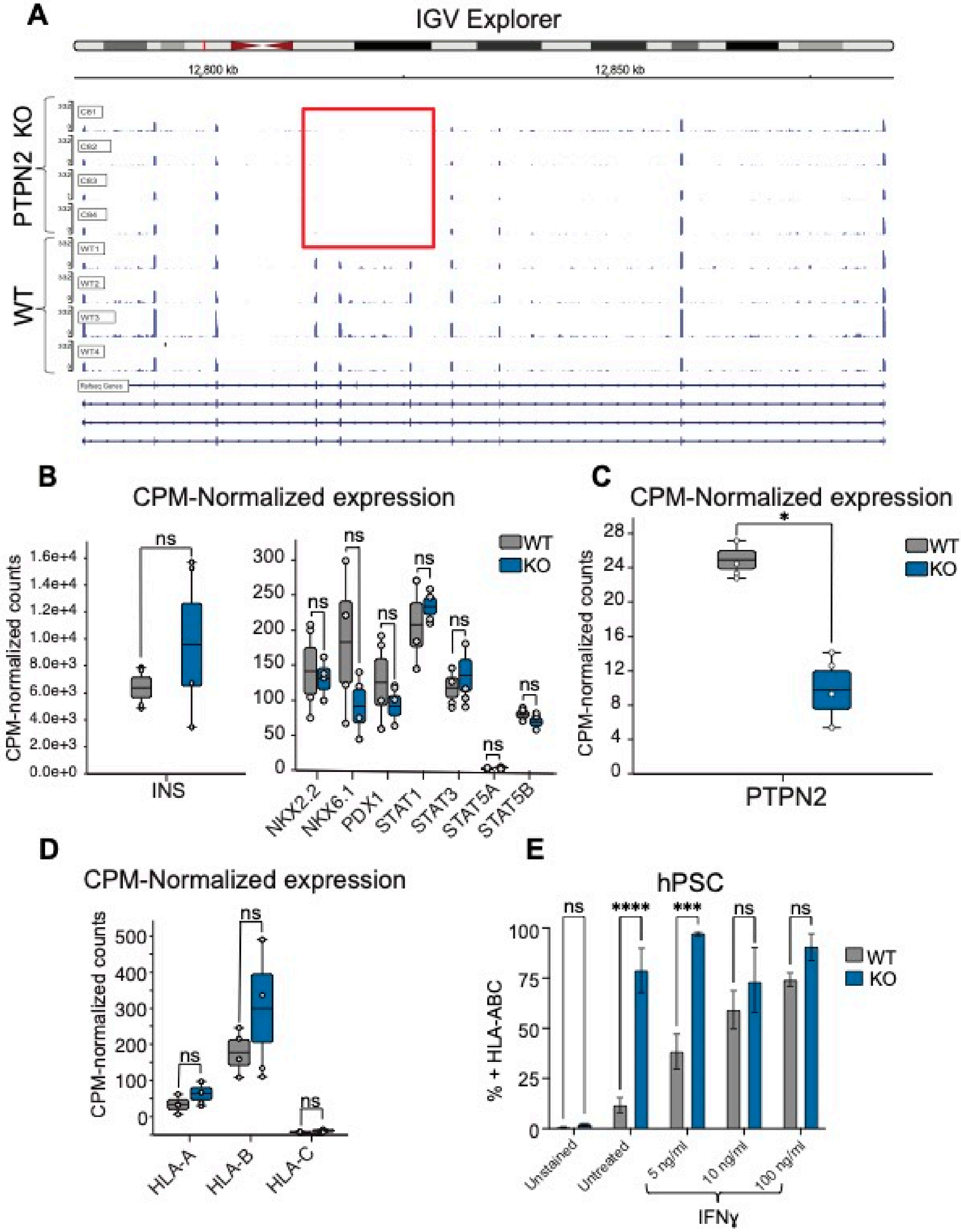

Supplemental Figure S3: RNAseq and HLA Class 1 expression in WT and PTPN2 KO cells

A: Bulk RNAseq IGV explorer from WT and PTPN2 KO GFP+ sBCs demonstrating lack of gene expression in exons 5-8 of the *PTPN2* region for PTPN2 KO compared to WT. B: Bar graph of the CPM-Normalized expression of *INS, NKX2.2, NKX6.1, PDX1, STAT1/3/5A/5B* on the y-axis with WT (gray) and PTPN2 KO (blue) GFP+ sBCs on the x-axis. Bars are drawn at the average value for each group and are colored according to group. Error bars represent the average +/- standard deviation (SD). non-significant=ns. C: Bar graph of the CPM-Normalized expression of *PTPN2* on the y-axis with WT (gray) and PTPN2 KO (blue) GFP+ sBCs on the x-axis. Bars are drawn at the average value for each group and are colored according to group. Error bars represent the average +/- standard deviation (SD). *p=0.003. D: Bar graph of the CPM-Normalized expression of *HLA-ABC* on the y-axis with WT (gray) and PTPN2 KO (blue) GFP+ sBCs on the x-axis. Bars are drawn at the average value for each group and are colored according to group. Error bars represent the average +/- standard deviation (SD). non-significant=ns. E: Flow cytometric quantification of percentage of HLA-ABC expression from WT (gray) and PTPN2 KO (blue) hPSC treated with and without IFNγ in increasing concentrations: from 5 ng/ml, 10 ng/ml and 100 ng/ml. (n=4) *** $p \leq 0.0002$, **** $p \leq 0.0001$; non-significant=ns.
